# Tri­chlorido­{meth­yl[4-(methyl­imino)­pent-2-en-2-yl]aza­nido-κ^2^ *N*,*N*′}hafnium(IV)

**DOI:** 10.1107/S1600536813022745

**Published:** 2013-08-21

**Authors:** Ken Ikeda, Hideki Masuda

**Affiliations:** a Department of Frontier Materials, Graduate School of Engineering, Nagoya Institute of Technology, Nagoya 466-8555, Japan

## Abstract

The title complex, [Hf(C_29_H_41_N_2_)Cl_3_], was synthesized from HfCl_4_ and HC[C(Me)N(^*i*^Pr_2_C_6_H_3_)]_2_Li(Et_2_O) in toluene. The structure is isotypic with those of Ti^IV^ and Zr^IV^ complexes reported previously [Nikiforov *et al.* (2007 ▶). *Dalton Trans*. pp. 4149–4159; Kakaliou *et al.* (1999 ▶). *Inorg. Chem*. **38**, 5964–5977]. There is a crystallographic mirror plane containing the Hf atom, one chloride ligand and the central diketiminate C atom. The Hf^IV^ ion has slightly distorted square-pyramidal geometry surrounded by two N atoms from the β-diketiminate ligand and by three Cl^−^ anions. Coordination bond lengths are Hf—N = 2.181 (3) Å, and Hf—Cl = 2.3148 (15) and 2.3727 (12) Å.

## Related literature

For complexes bearing *β*-diketiminate ligands, see: Bourget-Merle *et al.* (2002 ▶); Hamaki *et al.* (2006 ▶). For the synthesis of the precursor of the *β*-diketiminate in the title compound, see: Stender *et al.* (2001 ▶). For isotypic Ti^IV^ and Zr^IV^ complexes, see: Nikiforov *et al.* (2007 ▶) and Kakaliou *et al.* (1999 ▶), respectively.
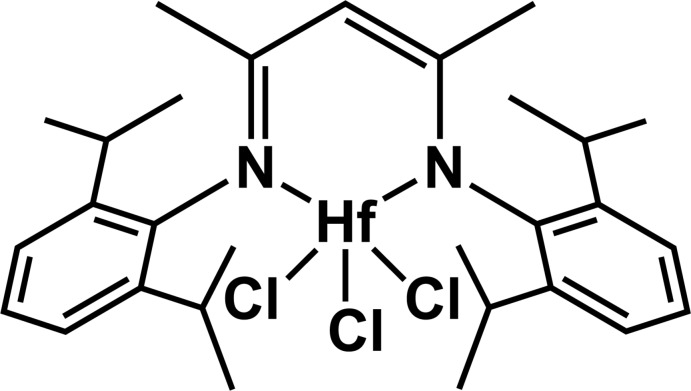


## Experimental

### 

#### Crystal data

[Hf(C_29_H_41_N_2_)Cl_3_]
*M* *_r_* = 702.51
Orthorhombic, 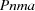

*a* = 13.980 (3) Å
*b* = 21.808 (4) Å
*c* = 9.8492 (16) Å
*V* = 3002.8 (9) Å^3^
*Z* = 4
Mo *K*α radiation
μ = 3.75 mm^−1^
*T* = 173 K
0.20 × 0.10 × 0.10 mm

#### Data collection

Rigaku Mercury70 diffractometer
22083 measured reflections
3527 independent reflections
3132 reflections with *F* ^2^ > 2σ(*F* ^2^)
*R*_int_ = 0.049

#### Refinement

*R*[*F* ^2^ > 2σ(*F* ^2^)] = 0.029
*wR*(*F* ^2^) = 0.068
*S* = 1.10
3527 reflections
163 parameters
H-atom parameters constrained
Δρ_max_ = 2.65 e Å^−3^
Δρ_min_ = −1.02 e Å^−3^

### 

Data collection: *CrystalClear* (Rigaku/MSC, 2006 ▶); cell refinement: *CrystalClear*; data reduction: *CrystalClear*; program(s) used to solve structure: *SHELXS97* (Sheldrick, 2008 ▶); program(s) used to refine structure: *SHELXL97* (Sheldrick, 2008 ▶); molecular graphics: *CrystalStructure* (Rigaku/MSC, 2006 ▶); software used to prepare material for publication: *CrystalStructure*.

## Supplementary Material

Crystal structure: contains datablock(s) General, I. DOI: 10.1107/S1600536813022745/bh2479sup1.cif

Structure factors: contains datablock(s) I. DOI: 10.1107/S1600536813022745/bh2479Isup2.hkl

Additional supplementary materials:  crystallographic information; 3D view; checkCIF report

## Figures and Tables

**Fig. 1.**
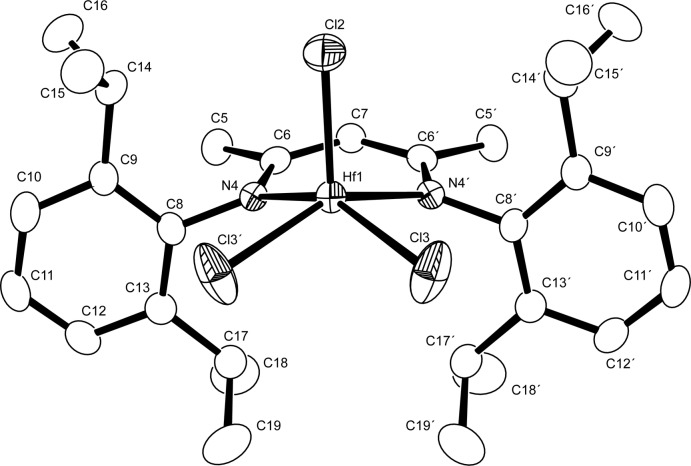
The molecular structure of the title molecule, with displacement ellipsoids at the 50% probability level.

## supplementary crystallographic information

### 1. Comment

Recently, both main element and transition metal complexes with bulky *β*-diketiminates as a supporting ligand have attracted considerable attention to small molecule activation (Bourget-Merle *et al.*, 2002). Especially, in the group 4, metal complexes of *β*-diketiminate were sometimes reported as pro-catalysts for the polymerization of olefins (Bourget-Merle *et al.*, 2002), nevertheless only one example of hafnium complex with a bulky *β*-diketiminate is known, to our best knowledge (Hamaki *et al.*, 2006). In this paper, we report a novel Hf(IV) complex with *β*-diketiminate, HC[C(Me)N(*^i^*Pr_2_C_6_H_3_)]_2_HfCl_3_. The central Hf(IV) ion has slightly distorted square-pyramidal geometry surrounded by two N atoms from *β*-diketiminate ligand and by three Cl^-^ anions. The selected geometric parameters are Hf—Cl2 = 2.3148 (15), Hf—Cl3 = 2.3727 (12), and Hf—N4 = 2.181 (3) Å. The reported complex is isomorphous to analogous complexes based on Titanium (Nikiforov *et al.*, 2007) and Zirconium (Kakaliou *et al.*, 1999).

### 2. Experimental

A solution of crude HC[C(Me)N(*^i^*Pr_2_C_6_H_3_)]_2_Li(Et_2_O) (2.0 g, 4.01 mmol, Stender *et al.*, 2001) in toluene (20 ml) was slowly added to HfCl_4_ (1.28 g, 4.01 mmol) and stirred overnight at room temperature. The solution was filtered through celite and the filtrate was concentrated under reduced pressure to afford a yellow solid. The yellow solid was extracted with *n*-pentane (20 ml) and cooled to -40 °C to yield yellow single crystals (1.52 g, 2.16 mmol, 54% yield).

### 3. Refinement

All H atoms were positioned geometrically and refined as riding atoms, with C—H = 0.95 (aromatic CH), 0.98 (methyl CH_3_) or 1.00 Å (methine CH), and *U*_iso_(H) = 1.2 *U*_eq_(carrier C).

### Crystal data

**Table:**

[Hf(C_29_H_41_N_2_)Cl_3_]	*F*(000) = 1408.00
*M**_r_* = 702.51	*D*_x_ = 1.554 Mg m^−^^3^
Orthorhombic, *P**n**m**a*	Mo *K*α radiation, λ = 0.71070 Å
Hall symbol: -P 2ac 2n	Cell parameters from 7615 reflections
*a* = 13.980 (3) Å	θ = 3.2–27.5°
*b* = 21.808 (4) Å	µ = 3.75 mm^−^^1^
*c* = 9.8492 (16) Å	*T* = 173 K
*V* = 3002.8 (9) Å^3^	Chip, yellow
*Z* = 4	0.20 × 0.10 × 0.10 mm

### Data collection

**Table:**

Rigaku Mercury70 diffractometer	*R*_int_ = 0.049
Detector resolution: 7.314 pixels mm^-1^	θ_max_ = 27.5°
ω scans	*h* = −18→18
22083 measured reflections	*k* = −28→23
3527 independent reflections	*l* = −12→12
3132 reflections with *F*^2^ > 2σ(*F*^2^)	

### Refinement

**Table:**

Refinement on *F*^2^	Primary atom site location: structure-invariant direct methods
*R*[*F*^2^ > 2σ(*F*^2^)] = 0.029	Secondary atom site location: difference Fourier map
*wR*(*F*^2^) = 0.068	Hydrogen site location: inferred from neighbouring sites
*S* = 1.10	H-atom parameters constrained
3527 reflections	*w* = 1/[σ^2^(*F*_o_^2^) + (0.0262*P*)^2^ + 3.927*P*] where *P* = (*F*_o_^2^ + 2*F*_c_^2^)/3
163 parameters	(Δ/σ)_max_ = 0.001
0 restraints	Δρ_max_ = 2.65 e Å^−^^3^
0 constraints	Δρ_min_ = −1.02 e Å^−^^3^

### Fractional atomic coordinates and isotropic or equivalent isotropic displacement parameters (Å^2^)

**Table:**

	*x*	*y*	*z*	*U*_iso_*/*U*_eq_	
Hf1	0.351205 (13)	0.2500	0.235799 (18)	0.02245 (7)	
Cl2	0.45574 (11)	0.2500	0.41806 (13)	0.0426 (4)	
Cl3	0.23188 (8)	0.17538 (5)	0.28104 (13)	0.0590 (4)	
N4	0.41607 (18)	0.31701 (12)	0.0996 (3)	0.0211 (6)	
C5	0.5517 (3)	0.36044 (15)	−0.0307 (4)	0.0324 (8)	
C6	0.4993 (3)	0.30737 (15)	0.0335 (3)	0.0241 (7)	
C7	0.5401 (3)	0.2500	0.0146 (4)	0.0245 (9)	
C8	0.3782 (3)	0.37893 (15)	0.0859 (4)	0.0237 (7)	
C9	0.4094 (3)	0.42552 (15)	0.1743 (4)	0.0263 (7)	
C10	0.3725 (3)	0.48427 (16)	0.1540 (4)	0.0337 (8)	
C11	0.3085 (3)	0.49681 (16)	0.0516 (4)	0.0355 (8)	
C12	0.2790 (3)	0.45035 (16)	−0.0334 (4)	0.0354 (9)	
C13	0.3121 (3)	0.39080 (15)	−0.0172 (4)	0.0288 (7)	
C14	0.4806 (3)	0.41495 (16)	0.2885 (4)	0.0317 (8)	
C15	0.4313 (3)	0.4205 (2)	0.4270 (4)	0.0454 (10)	
C16	0.5654 (3)	0.4587 (2)	0.2813 (5)	0.0466 (11)	
C17	0.2757 (3)	0.34099 (18)	−0.1115 (5)	0.0450 (11)	
C18	0.3140 (5)	0.3483 (3)	−0.2541 (5)	0.0707 (17)	
C19	0.1663 (4)	0.3385 (3)	−0.1144 (6)	0.0633 (14)	
H20B	0.5093	0.3813	−0.0950	0.0389*	
H21C	0.6083	0.3452	−0.0787	0.0389*	
H22A	0.5716	0.3893	0.0401	0.0389*	
H23	0.6047	0.2500	−0.0154	0.0294*	
H24	0.3922	0.5165	0.2125	0.0404*	
H25	0.2848	0.5373	0.0396	0.0426*	
H26	0.2352	0.4592	−0.1044	0.0425*	
H27	0.5057	0.3722	0.2798	0.0380*	
H28A	0.3783	0.3912	0.4320	0.0545*	
H29C	0.4776	0.4116	0.4991	0.0545*	
H30B	0.4066	0.4622	0.4384	0.0545*	
H31C	0.6139	0.4461	0.3476	0.0559*	
H32A	0.5439	0.5004	0.3019	0.0559*	
H33B	0.5930	0.4576	0.1898	0.0559*	
H34	0.2991	0.3008	−0.0758	0.0540*	
H35B	0.1417	0.3734	−0.1665	0.0760*	
H36A	0.1456	0.3002	−0.1570	0.0760*	
H37C	0.1416	0.3404	−0.0213	0.0760*	
H38A	0.2893	0.3151	−0.3113	0.0849*	
H39B	0.3840	0.3466	−0.2523	0.0849*	
H40C	0.2933	0.3878	−0.2910	0.0849*	

### Atomic displacement parameters (Å^2^)

**Table:**

	*U*^11^	*U*^22^	*U*^33^	*U*^12^	*U*^13^	*U*^23^
Hf1	0.02264 (11)	0.02380 (12)	0.02091 (11)	0.0000	0.00470 (7)	0.0000
Cl2	0.0552 (9)	0.0443 (8)	0.0284 (7)	0.0000	−0.0114 (6)	0.0000
Cl3	0.0439 (6)	0.0417 (6)	0.0914 (9)	−0.0175 (5)	0.0394 (6)	−0.0235 (6)
N4	0.0225 (12)	0.0220 (15)	0.0188 (13)	−0.0011 (11)	0.0019 (10)	−0.0003 (10)
C5	0.0320 (18)	0.0295 (19)	0.0358 (19)	−0.0063 (15)	0.0100 (15)	0.0021 (15)
C6	0.0233 (15)	0.0288 (17)	0.0201 (15)	−0.0045 (13)	0.0006 (12)	0.0006 (13)
C7	0.021 (2)	0.027 (3)	0.026 (3)	0.0000	0.0042 (17)	0.0000
C8	0.0251 (15)	0.0218 (17)	0.0242 (16)	0.0010 (13)	0.0063 (13)	0.0033 (13)
C9	0.0282 (17)	0.0245 (18)	0.0261 (17)	0.0020 (13)	0.0062 (13)	−0.0026 (14)
C10	0.0374 (19)	0.0248 (19)	0.039 (2)	−0.0017 (15)	0.0094 (16)	−0.0045 (15)
C11	0.0376 (19)	0.0228 (19)	0.046 (3)	0.0050 (15)	0.0081 (17)	0.0058 (16)
C12	0.0345 (19)	0.032 (2)	0.040 (2)	0.0035 (15)	−0.0027 (16)	0.0115 (16)
C13	0.0316 (17)	0.0270 (18)	0.0277 (17)	−0.0015 (14)	−0.0009 (14)	0.0050 (14)
C14	0.0343 (19)	0.0293 (19)	0.0314 (18)	−0.0014 (15)	−0.0009 (15)	−0.0084 (14)
C15	0.055 (3)	0.050 (3)	0.031 (2)	0.003 (2)	0.0006 (18)	−0.0088 (18)
C16	0.039 (3)	0.049 (3)	0.052 (3)	−0.011 (2)	−0.0043 (19)	−0.0146 (19)
C17	0.058 (3)	0.029 (2)	0.048 (3)	0.0022 (18)	−0.027 (2)	0.0018 (17)
C18	0.073 (4)	0.088 (5)	0.051 (3)	0.014 (4)	−0.014 (3)	−0.032 (3)
C19	0.068 (4)	0.061 (4)	0.060 (3)	−0.023 (3)	−0.025 (3)	0.005 (3)

### Geometric parameters (Å, º)

**Table:**

Hf1—Cl2	2.3148 (15)	C5—H20B	0.980
Hf1—Cl3	2.3727 (12)	C5—H21C	0.980
Hf1—Cl3^i^	2.3727 (12)	C5—H22A	0.980
Hf1—N4	2.181 (3)	C7—H23	0.950
Hf1—N4^i^	2.181 (3)	C10—H24	0.950
N4—C6	1.349 (4)	C11—H25	0.950
N4—C8	1.457 (5)	C12—H26	0.950
C5—C6	1.509 (5)	C14—H27	1.000
C6—C7	1.388 (4)	C15—H28A	0.980
C8—C9	1.407 (5)	C15—H29C	0.980
C8—C13	1.398 (5)	C15—H30B	0.980
C9—C10	1.395 (5)	C16—H31C	0.980
C9—C14	1.519 (5)	C16—H32A	0.980
C10—C11	1.376 (6)	C16—H33B	0.980
C11—C12	1.377 (6)	C17—H34	1.000
C12—C13	1.387 (5)	C18—H38A	0.980
C13—C17	1.517 (6)	C18—H39B	0.980
C14—C15	1.533 (6)	C18—H40C	0.980
C14—C16	1.523 (6)	C19—H35B	0.980
C17—C18	1.511 (7)	C19—H36A	0.980
C17—C19	1.531 (7)	C19—H37C	0.980
			
Hf1···C6^i^	3.134 (3)	H36A···H40C	3.1084
Hf1···C7	3.423 (5)	H37C···H38A	3.5678
Hf1···C8^i^	3.198 (4)	H37C···H40C	3.5528
Cl3···C8^i^	3.046 (4)	H38A···H38A^i^	2.8376
Cl3···C9^i^	3.480 (4)	Cl2···H37C^ii^	3.4172
Cl3···C13^i^	3.460 (4)	Cl2···H37C^iii^	3.4172
N4···C6^i^	3.022 (5)	Cl3···H21C^iv^	3.4324
N4···C14	2.973 (5)	Cl3···H22A^iv^	3.1806
N4···C17	2.906 (5)	Cl3···H23^iv^	3.3374
C5···C8	2.714 (5)	Cl3···H27^iv^	3.3816
C5···C9	3.169 (5)	Cl3···H31C^iv^	3.3684
C5···C13	3.418 (5)	Cl3···H33B^iv^	3.5015
C5···C14	3.505 (5)	C5···H24^v^	3.3202
C6···C9	3.184 (5)	C5···H25^v^	3.1951
C6···C13	3.226 (5)	C5···H35B^vi^	3.2492
C6···C14	3.447 (5)	C5···H36A^vi^	3.5932
C6···C17	3.514 (6)	C10···H20B^v^	3.4145
C8···C11	2.770 (5)	C10···H22A^v^	3.4442
C8···C15	3.558 (5)	C10···H26^vii^	3.0737
C8···C18	3.531 (6)	C10···H35B^vii^	3.5779
C9···C12	2.793 (5)	C11···H22A^v^	3.1289
C10···C13	2.778 (5)	C11···H26^vii^	3.5735
C10···C15	3.137 (6)	C11···H30B^viii^	3.3299
C10···C16	3.026 (6)	C11···H31C^ix^	3.0996
C12···C18	3.150 (7)	C11···H33B^v^	2.9226
C12···C19	3.011 (7)	C11···H40C^vii^	3.2803
Hf1···H27	3.4567	C12···H24^viii^	3.5377
Hf1···H27^i^	3.4567	C12···H30B^viii^	3.2326
Hf1···H34	3.3432	C12···H31C^ix^	2.9471
Hf1···H34^i^	3.3432	C12···H33B^v^	3.1000
Cl2···H27	3.0726	C13···H31C^ix^	3.4529
Cl2···H27^i^	3.0726	C15···H25^vii^	3.3478
Cl2···H28A	3.2664	C15···H26^vii^	3.5208
Cl2···H28A^i^	3.2664	C15···H32A^x^	3.1979
Cl3···H28A^i^	2.9170	C15···H37C^iii^	3.5433
Cl3···H37C^i^	3.2526	C15···H40C^xi^	3.4560
N4···H20B	2.7090	C16···H30B^x^	3.2793
N4···H21C	3.2680	C18···H21C^xii^	3.3144
N4···H22A	2.7492	C18···H25^viii^	3.5028
N4···H23	3.2201	C18···H28A^xiii^	3.3537
N4···H27	2.4832	C19···H21C^xii^	3.1334
N4···H34	2.4045	C19···H29C^ix^	3.2847
N4···H34^i^	3.5007	C19···H31C^ix^	3.5986
N4···H39B	3.5534	H20B···C10^v^	3.4145
C5···H23	2.5242	H20B···H24^v^	2.8635
C5···H27	3.1349	H20B···H25^v^	3.4260
C5···H33B	3.0880	H20B···H32A^v^	3.3703
C5···H39B	3.2181	H20B···H35B^vi^	2.9948
C6···H27	2.8088	H20B···H36A^vi^	3.5667
C6···H34	3.0015	H21C···Cl3^ii^	3.4324
C6···H39B	3.3543	H21C···C18^vi^	3.3144
C7···H20B	3.0904	H21C···C19^vi^	3.1334
C7···H20B^i^	3.0904	H21C···H24^v^	3.2920
C7···H21C	2.4616	H21C···H25^v^	2.9928
C7···H21C^i^	2.4616	H21C···H35B^vi^	2.6257
C7···H22A	3.0807	H21C···H36A^vi^	2.8300
C7···H22A^i^	3.0807	H21C···H38A^vi^	2.8303
C8···H20B	2.5573	H21C···H40C^vi^	3.0341
C8···H22A	2.7505	H22A···Cl3^ii^	3.1806
C8···H24	3.2554	H22A···C10^v^	3.4442
C8···H26	3.2523	H22A···C11^v^	3.1289
C8···H27	2.6160	H22A···H24^v^	3.2656
C8···H28A	3.4192	H22A···H25^v^	2.6850
C8···H34	2.5814	H23···Cl3^ii^	3.3374
C8···H37C	3.5718	H23···Cl3^iii^	3.3374
C8···H39B	3.4060	H23···H36A^xiv^	3.4545
C9···H20B	3.1492	H23···H36A^vi^	3.4545
C9···H22A	2.7401	H23···H38A^xiv^	3.4045
C9···H25	3.2766	H23···H38A^vi^	3.4045
C9···H28A	2.6818	H24···C5^v^	3.3202
C9···H29C	3.3521	H24···C12^vii^	3.5377
C9···H30B	2.7215	H24···H20B^v^	2.8635
C9···H31C	3.3598	H24···H21C^v^	3.2920
C9···H32A	2.7896	H24···H22A^v^	3.2656
C9···H33B	2.6637	H24···H26^vii^	2.5889
C10···H26	3.2347	H24···H35B^vii^	2.7218
C10···H27	3.3127	H24···H40C^vii^	3.3290
C10···H28A	3.4094	H25···C5^v^	3.1951
C10···H30B	2.8813	H25···C15^viii^	3.3478
C10···H32A	2.8258	H25···C18^vii^	3.5028
C10···H33B	3.1557	H25···H20B^v^	3.4260
C12···H24	3.2331	H25···H21C^v^	2.9928
C12···H34	3.3005	H25···H22A^v^	2.6850
C12···H35B	2.8678	H25···H26^vii^	3.5183
C12···H37C	3.0741	H25···H28A^viii^	2.9593
C12···H39B	3.4533	H25···H30B^viii^	2.8558
C12···H40C	2.8877	H25···H31C^ix^	3.3002
C13···H20B	2.8696	H25···H33B^v^	2.8353
C13···H25	3.2652	H25···H40C^vii^	2.5780
C13···H35B	2.8250	H26···C10^viii^	3.0737
C13···H36A	3.3496	H26···C11^viii^	3.5735
C13···H37C	2.6241	H26···C15^viii^	3.5208
C13···H38A	3.3496	H26···H24^viii^	2.5889
C13···H39B	2.7022	H26···H25^viii^	3.5183
C13···H40C	2.7100	H26···H30B^viii^	2.6539
C14···H22A	2.8131	H26···H31C^ix^	3.0590
C14···H24	2.6445	H26···H33B^v^	3.1254
C15···H24	3.0241	H27···Cl3^ii^	3.3816
C15···H31C	2.7278	H27···H37C^iii^	3.1229
C15···H32A	2.6519	H28A···C18^xi^	3.3537
C15···H33B	3.3488	H28A···H25^vii^	2.9593
C16···H22A	2.8170	H28A···H38A^xi^	3.2702
C16···H24	2.8133	H28A···H39B^xi^	3.2591
C16···H28A	3.3482	H28A···H40C^xi^	2.9765
C16···H29C	2.6767	H29C···C19^iii^	3.2847
C16···H30B	2.7069	H29C···H30B^x^	3.2514
C17···H20B	3.3862	H29C···H32A^x^	2.7591
C17···H26	2.6413	H29C···H35B^iii^	2.9453
C17···H34^i^	3.1288	H29C···H37C^iii^	2.7779
C18···H20B	3.2300	H29C···H39B^xi^	3.1172
C18···H26	3.0403	H29C···H40C^xi^	3.3429
C18···H35B	2.6163	H30B···C11^vii^	3.3299
C18···H36A	2.7483	H30B···C12^vii^	3.2326
C18···H37C	3.3298	H30B···C16^x^	3.2793
C19···H26	2.8053	H30B···H25^vii^	2.8558
C19···H34^i^	3.5806	H30B···H26^vii^	2.6539
C19···H36A^i^	3.0673	H30B···H29C^x^	3.2514
C19···H38A	2.6427	H30B···H30B^x^	3.3174
C19···H39B	3.3372	H30B···H31C^x^	2.9193
C19···H40C	2.7087	H30B···H32A^x^	2.7726
H20B···H23	3.2545	H30B···H40C^xi^	3.4993
H20B···H33B	3.4651	H31C···Cl3^ii^	3.3684
H20B···H34	3.4284	H31C···C11^iii^	3.0996
H20B···H39B	2.4584	H31C···C12^iii^	2.9471
H20B···H40C	3.5865	H31C···C13^iii^	3.4529
H21C···H23	2.1675	H31C···C19^iii^	3.5986
H21C···H39B	3.5718	H31C···H25^iii^	3.3002
H22A···H23	3.1219	H31C···H26^iii^	3.0590
H22A···H27	2.5613	H31C···H30B^x^	2.9193
H22A···H31C	3.3247	H31C···H35B^iii^	3.5407
H22A···H32A	3.5586	H31C···H37C^iii^	2.8971
H22A···H33B	2.1161	H32A···C15^x^	3.1979
H24···H25	2.3158	H32A···H20B^v^	3.3703
H24···H27	3.5866	H32A···H29C^x^	2.7591
H24···H28A	3.4900	H32A···H30B^x^	2.7726
H24···H30B	2.5277	H32A···H39B^v^	3.5202
H24···H32A	2.3230	H32A···H40C^v^	3.3360
H24···H33B	3.0951	H33B···Cl3^ii^	3.5015
H25···H26	2.3209	H33B···C11^v^	2.9226
H26···H34	3.5806	H33B···C12^v^	3.1000
H26···H35B	2.3646	H33B···H25^v^	2.8353
H26···H37C	3.0160	H33B···H26^v^	3.1254
H26···H39B	3.5336	H35B···C5^xii^	3.2492
H26···H40C	2.5425	H35B···C10^viii^	3.5779
H27···H28A	2.3643	H35B···H20B^xii^	2.9948
H27···H29C	2.3585	H35B···H21C^xii^	2.6257
H27···H30B	2.8660	H35B···H24^viii^	2.7218
H27···H31C	2.3106	H35B···H29C^ix^	2.9453
H27···H32A	2.8556	H35B···H31C^ix^	3.5407
H27···H33B	2.3963	H36A···C5^xii^	3.5932
H28A···H32A	3.5603	H36A···H20B^xii^	3.5667
H29C···H31C	2.5357	H36A···H21C^xii^	2.8300
H29C···H32A	2.8958	H36A···H23^xv^	3.4545
H29C···H33B	3.5899	H37C···Cl2^iv^	3.4172
H30B···H31C	3.0532	H37C···C15^ix^	3.5433
H30B···H32A	2.4868	H37C···H27^ix^	3.1229
H30B···H33B	3.5759	H37C···H29C^ix^	2.7779
H34···H34^i^	2.2148	H37C···H31C^ix^	2.8971
H34···H35B	2.8549	H38A···H21C^xii^	2.8303
H34···H36A	2.2909	H38A···H23^xv^	3.4045
H34···H36A^i^	3.1772	H38A···H28A^xiii^	3.2702
H34···H37C	2.4257	H39B···H28A^xiii^	3.2591
H34···H38A	2.3444	H39B···H29C^xiii^	3.1172
H34···H38A^i^	3.4323	H39B···H32A^v^	3.5202
H34···H39B	2.3292	H40C···C11^viii^	3.2803
H34···H40C	2.8463	H40C···C15^xiii^	3.4560
H35B···H38A	2.8131	H40C···H21C^xii^	3.0341
H35B···H39B	3.5400	H40C···H24^viii^	3.3290
H35B···H40C	2.4696	H40C···H25^viii^	2.5780
H36A···H36A^i^	2.1882	H40C···H28A^xiii^	2.9765
H36A···H37C^i^	3.3453	H40C···H29C^xiii^	3.3429
H36A···H38A	2.5404	H40C···H30B^xiii^	3.4993
H36A···H38A^i^	3.5585	H40C···H32A^v^	3.3360
			
Cl2—Hf1—Cl3	107.35 (4)	H21C—C5—H22A	109.466
Cl2—Hf1—Cl3^i^	107.35 (4)	C6—C7—H23	115.628
Cl2—Hf1—N4	102.39 (8)	C6^i^—C7—H23	115.628
Cl2—Hf1—N4^i^	102.39 (8)	C9—C10—H24	119.072
Cl3—Hf1—Cl3^i^	86.61 (4)	C11—C10—H24	119.075
Cl3—Hf1—N4	150.14 (8)	C10—C11—H25	120.198
Cl3—Hf1—N4^i^	87.04 (8)	C12—C11—H25	120.205
Cl3^i^—Hf1—N4	87.04 (8)	C11—C12—H26	119.356
Cl3^i^—Hf1—N4^i^	150.14 (8)	C13—C12—H26	119.357
N4—Hf1—N4^i^	84.12 (10)	C9—C14—H27	107.891
Hf1—N4—C6	123.4 (2)	C15—C14—H27	107.901
Hf1—N4—C8	121.8 (2)	C16—C14—H27	107.895
C6—N4—C8	114.4 (3)	C14—C15—H28A	109.471
N4—C6—C5	120.1 (3)	C14—C15—H29C	109.465
N4—C6—C7	124.0 (3)	C14—C15—H30B	109.478
C5—C6—C7	115.8 (3)	H28A—C15—H29C	109.472
C6—C7—C6^i^	128.7 (4)	H28A—C15—H30B	109.472
N4—C8—C9	120.0 (3)	H29C—C15—H30B	109.470
N4—C8—C13	118.6 (3)	C14—C16—H31C	109.474
C9—C8—C13	121.4 (3)	C14—C16—H32A	109.459
C8—C9—C10	117.4 (3)	C14—C16—H33B	109.477
C8—C9—C14	123.5 (3)	H31C—C16—H32A	109.468
C10—C9—C14	119.2 (3)	H31C—C16—H33B	109.482
C9—C10—C11	121.9 (4)	H32A—C16—H33B	109.467
C10—C11—C12	119.6 (4)	C13—C17—H34	107.625
C11—C12—C13	121.3 (4)	C18—C17—H34	107.627
C8—C13—C12	118.5 (3)	C19—C17—H34	107.628
C8—C13—C17	122.3 (3)	C17—C18—H38A	109.459
C12—C13—C17	119.2 (4)	C17—C18—H39B	109.471
C9—C14—C15	110.6 (3)	C17—C18—H40C	109.474
C9—C14—C16	112.4 (3)	H38A—C18—H39B	109.469
C15—C14—C16	110.0 (3)	H38A—C18—H40C	109.473
C13—C17—C18	112.0 (4)	H39B—C18—H40C	109.481
C13—C17—C19	111.8 (4)	C17—C19—H35B	109.477
C18—C17—C19	109.9 (4)	C17—C19—H36A	109.473
C6—C5—H20B	109.467	C17—C19—H37C	109.470
C6—C5—H21C	109.476	H35B—C19—H36A	109.468
C6—C5—H22A	109.471	H35B—C19—H37C	109.471
H20B—C5—H21C	109.476	H36A—C19—H37C	109.467
H20B—C5—H22A	109.470		
			
Cl2—Hf1—N4—C6	69.28 (18)	N4—C6—C7—C6^i^	13.4 (6)
Cl2—Hf1—N4—C8	−103.09 (16)	C5—C6—C7—C6^i^	−164.0 (4)
Cl2—Hf1—N4^i^—C6^i^	−69.28 (18)	N4—C8—C9—C10	178.3 (3)
Cl2—Hf1—N4^i^—C8^i^	103.09 (16)	N4—C8—C9—C14	−1.4 (5)
Cl3—Hf1—N4—C6	−105.67 (19)	N4—C8—C13—C12	−177.5 (3)
Cl3—Hf1—N4—C8	82.0 (3)	N4—C8—C13—C17	2.4 (5)
Cl3—Hf1—N4^i^—C6^i^	−176.40 (18)	C9—C8—C13—C12	1.5 (5)
Cl3—Hf1—N4^i^—C8^i^	−4.03 (16)	C9—C8—C13—C17	−178.6 (3)
Cl3^i^—Hf1—N4—C6	176.40 (18)	C13—C8—C9—C10	−0.7 (5)
Cl3^i^—Hf1—N4—C8	4.03 (16)	C13—C8—C9—C14	179.6 (3)
Cl3^i^—Hf1—N4^i^—C6^i^	105.67 (19)	C8—C9—C10—C11	−0.3 (5)
Cl3^i^—Hf1—N4^i^—C8^i^	−82.0 (3)	C8—C9—C14—C15	−110.5 (4)
N4—Hf1—N4^i^—C6^i^	32.15 (18)	C8—C9—C14—C16	126.1 (3)
N4—Hf1—N4^i^—C8^i^	−155.47 (18)	C10—C9—C14—C15	69.8 (4)
N4^i^—Hf1—N4—C6	−32.15 (18)	C10—C9—C14—C16	−53.6 (4)
N4^i^—Hf1—N4—C8	155.47 (18)	C14—C9—C10—C11	179.4 (3)
Hf1—N4—C6—C5	−164.94 (16)	C9—C10—C11—C12	0.4 (6)
Hf1—N4—C6—C7	17.8 (4)	C10—C11—C12—C13	0.5 (6)
Hf1—N4—C8—C9	87.6 (3)	C11—C12—C13—C8	−1.5 (5)
Hf1—N4—C8—C13	−93.4 (3)	C11—C12—C13—C17	178.6 (3)
C6—N4—C8—C9	−85.4 (4)	C8—C13—C17—C18	−109.4 (4)
C6—N4—C8—C13	93.6 (4)	C8—C13—C17—C19	126.7 (4)
C8—N4—C6—C5	7.9 (4)	C12—C13—C17—C18	70.5 (5)
C8—N4—C6—C7	−169.3 (3)	C12—C13—C17—C19	−53.4 (5)

Symmetry codes: (i) *x*, −*y*+1/2, *z*; (ii) *x*+1/2, −*y*+1/2, −*z*+1/2; (iii) *x*+1/2, *y*, −*z*+1/2; (iv) *x*−1/2, −*y*+1/2, −*z*+1/2; (v) −*x*+1, −*y*+1, −*z*; (vi) *x*+1/2, *y*, −*z*−1/2; (vii) −*x*+1/2, −*y*+1, *z*+1/2; (viii) −*x*+1/2, −*y*+1, *z*−1/2; (ix) *x*−1/2, *y*, −*z*+1/2; (x) −*x*+1, −*y*+1, −*z*+1; (xi) *x*, *y*, *z*+1; (xii) *x*−1/2, *y*, −*z*−1/2; (xiii) *x*, *y*, *z*−1; (xiv) *x*+1/2, −*y*+1/2, −*z*−1/2; (xv) *x*−1/2, −*y*+1/2, −*z*−1/2.
